# TGF-β signaling is an effective target to impair survival and induce apoptosis of human cholangiocarcinoma cells: A study on human primary cell cultures

**DOI:** 10.1371/journal.pone.0183932

**Published:** 2017-09-05

**Authors:** Anna Maria Lustri, Sabina Di Matteo, Alice Fraveto, Daniele Costantini, Alfredo Cantafora, Chiara Napoletano, Maria Consiglia Bragazzi, Felice Giuliante, Agostino M. De Rose, Pasquale B. Berloco, Gian Luca Grazi, Guido Carpino, Domenico Alvaro

**Affiliations:** 1 Medico-surgical Sciences and Biotechnologies, Sapienza University of Rome, RM, ROMA, Italy; 2 Department of Experimental Medicine, University of Rome Sapienza, Roma, Italy; 3 Catholic University of the Sacred Heart School of Medicine, Roma, Italy; 4 Department of General Surgery and Organ Transplantation, Sapienza University of Rome, Roma, Italy; 5 Regina Elena National Cancer Institute, the Gastroenterology Unit, Roma, Italy; 6 Department of Health Science, University of Rome Foro Italico, Roma, Italy; 7 Department of Internal Medicine and Medical Specialties, Sapienza University of Rome, RM, ROMA, Italy; University of South Alabama Mitchell Cancer Institute, UNITED STATES

## Abstract

Cholangiocarcinoma (CCA) and its subtypes (mucin- and mixed-CCA) arise from the neoplastic transformation of cholangiocytes, the epithelial cells lining the biliary tree. CCA has a high mortality rate owing to its aggressiveness, late diagnosis and high resistance to radiotherapy and chemotherapeutics. We have demonstrated that CCA is enriched for cancer stem cells which express epithelial to mesenchymal transition (EMT) traits, with these features being associated with aggressiveness and drug resistance. TGF-β signaling is upregulated in CCA and involved in EMT. We have recently established primary cell cultures from human mucin- and mixed-intrahepatic CCA. In human CCA primary cultures with different levels of EMT trait expression, we evaluated the anticancer effects of: (i) CX-4945, a casein kinase-2 (CK2) inhibitor that blocks TGF-β1-induced EMT; and (ii) LY2157299, a TGF-β receptor I kinase inhibitor. We tested primary cell lines expressing EMT trait markers (vimentin, N-cadherin and nuclear catenin) but negative for epithelial markers, and cell lines expressing epithelial markers (CK19-positive) in association with EMT traits. Cell viability was evaluated by MTS assays, apoptosis by Annexin V FITC and cell migration by wound-healing assay. Results: at a dose of 10 μM, CX4945 significantly decreased cell viability of primary human cell cultures from both mucin and mixed CCA, whereas in CK19-positive cell cultures, the effect of CX4945 on cell viability required higher concentrations (>30μM). At the same concentrations, CX4945 also induced apoptosis (3- fold increase vs controls) which correlated with the expression level of CK2 in the different CCA cell lines (mucin- and mixed-CCA). Indeed, no apoptotic effects were observed in CK19-positive cells expressing lower CK2 levels. The effects of CX4945 on viability and apoptosis were associated with an increased number of γ-H2ax (biomarker for DNA double-strand breaks) foci, suggesting the active role of CK2 as a repair mechanism in CCAs. LY2157299 failed to influence cell proliferation or apoptosis but significantly inhibited cell migration. At a 50 μM concentration, in fact, LY2157299 significantly impaired (at 24, 48 and 120 hrs) the wound-healing of primary cell cultures from both mucin-and mixed-CCA. In conclusion, we demonstrated that CX4945 and LY2157299 exert relevant but distinct anticancer effects against human CCA cells, with CX4945 acting on cell viability and apoptosis, and LY2157299 impairing cell migration. These results suggest that targeting the TGF-β signaling with a combination of CX-4945 and LY2157299 could have potential benefits in the treatment of human CCA.

## Introduction

Cholangiocarcinoma (CCA) is a heterogonous cancer originating from the neoplastic transformation of the epithelial cells lining the intrahepatic or extrahepatic biliary tree and associated peribiliary glands [1]. CCA is currently classified as intrahepatic (IHCCA), perihilar (pCCA), or distal (dCCA) [Refs. EASL guide lines; 1]. Histological pCCA and dCCA are invariably pure-mucin secreting adenocarcinoma, while, IH-CCA includes two different subtypes, a mucin-IHCCA similar to pCCA and a mixed-IHCCA in which areas of hepatocytic differentiation and neoplastic ductular reaction are also included within the tumor mass The two different subtypes of IHCCA likely originated from different cells, specifically the mucin-secreting epithelial cells lining large ducts and peribiliary glands in mucin-IHCCA, or the cuboidal non-mucin-secreting cells lining bile ductules or canals of Hering in mixed-IHCCA [2,3]. We have recently demonstrated how mixed-and mucin-IHCCA display a different profile of cancer stem cells (CSC) and a different sensitivity to chemotherapeutics or targeted agents with relevant implications for their clinical management [4, 5].

A number of recent evidence indicates that epithelial mesenchymal transition (EMT) is a key process for tumor progression, spreading and prognosis [6,7]. The EMT process implicates the epithelial cells to lose their junctions and apical–basal polarity and acquire the typical characteristics of mesenchymal cells [8,9]. This cellular process mainly occurs in the advanced phases of cancer development and involves TGFβ, a key member of the transforming growth factor family [10]. In the early phases of cancerogenesis, TGFβ displays pro-apoptotic effects which are abolished in intermediate phases as a consequence of the activation of oncogenic pathways such as Ras-MAPK, PI3K/AKT and c-Myc [11]. Overall, the complex of TGF-β/TGF-β receptor type II (TGF-ΒR2)/TGF-β receptor type I (TGF-ΒR1) induces Smad-dependent and-independent pathways which in turn drive EMT remodeling [12–14].

Different studies showed how EMT plays a key role in the progression of CCA, a cancer which demonstrates a typical desmoplastic nature and one in which the mesenchymal component predominates over the epithelial. In CCA, TGF-β, without affecting cell proliferation, drives cell migration [15, 16] by inducing the switch from the epithelial to the mesenchymal cell phenotype, which characterizes the EMT process. Consistently, TGF-β gene expression correlates with CCA prognosis as demonstrated by different reports [17]. In different cancer types, protein kinase CK2 is recruited by TGF-β-dependent pathways and modulates proliferation and EMT [18].

In this study, performed in primary cell cultures of human mucin- and mixed-IHCCA subtypes, we tried to counteract EMT by triggering TGF-β signaling and CK2. Selective inhibitors of TGF-β receptor type I (TGF-ΒR1) and CK2, already in clinical phases for different cancers, were tested for their anticancer effects in CCA.

## Materials and methods

### Primary cell cultures

The use of human materials was approved by our local Institutional Review Board. Primary cell cultures were prepared, as described [5], from specimens of human IHCCA obtained from patients submitted to surgical resection and classified as mucin- or mixed-IHCCA by morphologic criteria and Pas staining, according to Komuta M. et al [3]. CCA cell cultures were maintained in hormonally supplemented medium consisting of Dulbecco’s Modified Eagle Medium (DMEM) with high glucose/DMEM:F12 Nutrient mixture (1:1) (Gibco/BRL, Life Technologies, Italy srl., Milan, Italy) supplemented with 1.8 x 10^−4^ mol/L adenine, 5 μg/ml insulin, 5 μg/ml transferrin, 2 x 10^−9^ mol/L triiodothyronine, 1.7 x 10^−6^ mol/L hydrocortisone, 1.0 x 10^−6^ mol/L human epidermal growth factor, 5.5 x 10^−6^ mol/L epinephrine (Sigma-Aldrich, Milan, Italy), 10% fetal bovine serum (Gibco/BRL, Life Technologies, Milan, Italy), 100 U/ml of penicillin, and 100 μg/ml of streptomycin, at 37°C in a humidified atmosphere of 5% CO^2^ in air. Primary cell cultures were maintained at 37°C in a humidified atmosphere of 5% CO^2^ in air. Freshly isolated human biliary tree stem/progenitor cells (hBTSCs), obtained from the human biliary tree as recently described by Nevi et al. [19] were used as non neoplastic control cells. In all experiments, mucin- and mixed-IHCCA primary cells were tested after 20–30 passages [5], whereas Ck19-mucin was tested after 10–15 passages.

### Drugs

CX4945 (5-(3-chlorophenylamino)benzo[c][2,6]naphthyridine-8-carboxylic acid)) was purchased from Santa Cruz Biotechnology (Dallas, Texas, USA). LY2157299 and MK2206 were purchased from Selleck Chemicals (Houston, Texas, USA). All inhibitors were first dissolved in DMSO as stock solution and then added to the cell culture media at a dilution > 1: 10,000. The same aliquot of DMSO was added in untreated controls.

### Immunofluorescence

The primary cell culture was fixed in 1:1 Acetone / Methanol for 10 minutes, blocked in 20% fetal bovine serum (FBS) in Dulbecco’s Phosphate Buffered Saline (DPBS) (for 30 minute and then exposed for 1 hr at room temperature to anti-Vimentin sc-32322 (Santa Cruz Biotechnology, Dallas, Texas, USA), anti-Cytokeratin 19 (sc-6278, Santa Cruz Biotechnology, Dallas, Texas, USA), anti-Vimentin antibody RV202-FITCH (sc-32322 Santa Cruz Biotechnology, Dallas, Texas, USA) or mouse Anti-β-Catenin (cod. 610153, BD Pharmigen, Milan, Italy). Goat anti-Mouse IgG1 Texas Red (sc-2979, Santa Cruz Biotechnology, Dallas, Texas, USA) and Goat Anti-Mouse IgG2a Texas Red (sc-2980 Santa Cruz Biotechnology, Dallas, Texas, USA) were added for 1 hr at room temperature. For IF analysis of N-cadherin (sc-7939, Santa Cruz Biotechnology Dallas, Texas, USA), primary cell cultures were fixed in formalin 4% in DPBS (10 min. room temperature) and blocked (30 min.) in glycine (1M) and 30 min. in 20% FBS DPBS; permeabilization of cell membranes was performed with 1% bovine serum albumin (BSA) and 0.25% TRITON X-100 in DPBS for 10 min. Cells were then incubated with bovine anti-rabbit IgG Texas Red sc-2787 (Santa Cruz Biotechnology, Dallas, Texas, USA), for 1 hr at RT. For IF analysis of γH2ax (#9718, Cell Signaling, Danvers, MA, USA), cells were fixed in paraformaldehyde 4% in DPBS for 10 min at RT, blocked 1 hr in 5% FBS and 0.3% TRITON X-100 in DPBS. Cells were then incubated with bovine anti-rabbit IgG Texas Red sc-2787 (Santa Cruz Biotechnology Dallas, Texas) for 1 hr at room temperature. The fluorescence intensity was measured by using Image J. The γH2ax signal intensity in the nucleus was normalized to the DNA signal (DAPI) [20]. Results were expressed as the ratio of fluorescence intensity between treated and control cells. The nuclei were stained with 0.2μg/ml di 4,6-diamidino-2-phenylindole (DAPI, Sigma) for 2 min. at room temperature.

### RT-PCR

Total RNA was extracted from cell cultures by using the TRI REAGENT (Sigma-Aldrich, St Louis, MO, USA) and 1-bromo-3-chloropropane in substitution of chloroform. The reverse transcription primed by the random hexamer (Invitrogen s.r.l., S. Giuliano Milanese, Italy) was conducted in a 20 μL volume with 500 ngr of isolated RNA and the M-MLV reverse transcriptase (Invitrogen s.r.l.) according the manufacturer’s instructions. Gene expression was determined by Real-Time PCR with an MX3000P instrument (Agilent, La Jolla, CA, USA) using the average cycle threshold (Ct) automatically computed by the built-in software from three replicas of each sample. PCR amplifications were conducted in a volume of 25 μl, with 0.5 μl of cDNA template, 12.5 μl of 2x SYBR Green Brilliant QPCR Master Mix (Stratagene), 3 pmoles each of upstream and downstream primer for the gene analyzed, and 0.3 μl of diluted reference dye (ROX at a final concentration 30 nM). All real-time PCR amplifications were conducted with the cycling program: 10 min at 95°C followed by 40 cycles (30 sec at 95°C, 30 sec at 58°C, 30 sec at 72°C). The fluorescence detection was performed during the extension step of each cycle. The following genes of interest (GOI) were measured: TGF-ΒR1, CK2α, snail and vimentin. All expression levels were normalized with respect to hBTSCs, considered equal to 1. Table 1 shows the details of primers used in the study.

**Table 1:** Sequences of primer pairs used for amplifying the genes of interest (GOI) and the internal reference gene (GAPDH). Primers were designed by the PROBEFINDER software (https://www.roche-applied-science.com/sis/rtpcr/upl/index.jsp).

Gene Messenger	Primer Pair (5’->3’)	Length (nt)	Amplicon (bp)
GAPDH NM_002046	- F-AGCCACATCGCTCAGACAC	- 19	66
	- R-GCCCAATACGACCAAATCC	- 19	
TGFBR1 NM_004612.2	- F-GCA GAC TTA GGA CTG GCA GTA AG	- 23	104
	- R-AGA ACT TCA GGG GCC ATG T	- 19	
CK2 NM_177559.2	- F-GGT TGT ATG CTG GCA AGT ATG AT	- 23	90
	- R-AAC CTT GGC TAT CCT CAC CA	- 20	
VIMENTIN NM_003380	- F-CTGCCAACCGGAACAATGA	- 19	56
	- R-GTACTCAGTGGACTCCTGCTTT	- 22	
SNAIL NM_005985.3	- F-GCTGCAGGACTCTAATCCGA	- 21	84
	- R-ATCTCCGGAGGTGGGATG	- 18	

### MTS assay

Cell viability was evaluated by MTS assay (CellTiter 96 Aqueous One Solution, PROMEGA, Milan, Italy). A total of 3 x 10^3^ cells were seeded into 96-well plates in 100 μL of culture medium. After 24 hrs from seeding, the medium was replaced with fresh culture medium containing an increased concentration of the tested drug. The MTS assay was performed after an additional 72 hrs of exposure to each drug. The medium was not changed. Results were expressed as % changes with respect to controls considered equal to 1.

### Apoptosis assays

Apoptosis was measured, after 72 hrs of cell exposure to different drugs, by staining with BD Pharmingen kit, including FITCH Annexin V used in conjunction with a vital dye propidium iodide (PI) to identify late apoptotic cells (i.e. FITC Annexin and PI positive). The cells were analyzed by a BD FACSCantoTM Flow Cytometer (Becton, Dickinson and Company, NJ, USA). Ten thousand events were acquired and analyzed by BD FACSDiva software (Becton, Dickinson and Company, NJ, USA). Results were expressed as % of apoptotic cells.

### Wound healing assay

Mono-layers obtained by culture expansion until cell confluence were wounded by scraping with a 1000 μl pipette tip. The drugs were added after the scratches and maintained constantly for 120hrs, without changing the medium. The cells were monitored at intervals of 24h and pictures were collected at regular time intervals by Nikon Camera. The wound healing of treated cells (see before) was compared with untreated controls. We analyzed the migration data by Photoshop program. We calculated the ratio between the free area in T_0_ condition and in T_48h_ or T_120h_ condition.

### Clonogenic assay

Mucin-IHCCA cells were pre-treated for 72hrs with 10μM CX4945 or DMSO (CX4945-carrier, control) and subsequently re-plated in 24-well plates at a density of 200 cells/0.5ml for 8 days. Thereafter the medium was replaced and cells underwent treatments with MK2206 5μM or MK2206 5μM +CX4945 10μM or DMSO (control) for an additional two days. Colonies were counted to perform a clonogenic assay; a colony was considered a cluster of at least 50 cells. In addition, the clonogenicity was evaluated by measuring crystal violet staining (0.1% in ethanol). The excess dye was removed by washing and the absorbance of the extracted dye at 595 nm was measured.

### Phospho-RTK array analysis

Phospho-Receptor Tyrosine Kinase (RTK) array analysis was performed by a human phospho-RTK array kit (R&D Systems, #ARY001B, Minneapolis, MN), according to the manufacturer’s instructions. Mucin-IHCCA cells were seeded in a 100-mm culture dish at 5x10^5^. After 24h of plating, primary cell cultures were treated with CX4945 (10 μM) for 72 hrs. The cell lysates were obtained and, after blocking the reaction for 1 hr with the Array Buffer 1, 250 μg of protein lysates were incubated with membranes overnight at 4°C. Lysates were then washed and incubated with an HRP-conjugated phospho-tyrosine detection antibody before detection by chemiluminescence.

### Ethics statements

The research protocol was reviewed and approved by the Ethics Committee of Hospital Policlinico Umberto I of Rome/Sapienza University of Rome (full name of the board/committee; Prot. May 2014), and was conducted according to the principles expressed in the Declaration of Helsinki. Subjects have been properly instructed and have indicated that they consent to participate by signing the appropriate informed consent paperwork.

### Statistical analysis

Results are reported as means +/- standard error (SE). Statistical significance of difference between mean values was assessed using Sigma plot or the analysis of variance when multiple comparisons were performed. A p value < 0.05 was considered significant.

## Results

### Characterization of primary IHCCA cell cultures

Primary cell cultures prepared from surgical specimens of mucin- and mixed-IHCCA were analyzed by IF and RT-PCR.

**Fig 1:**
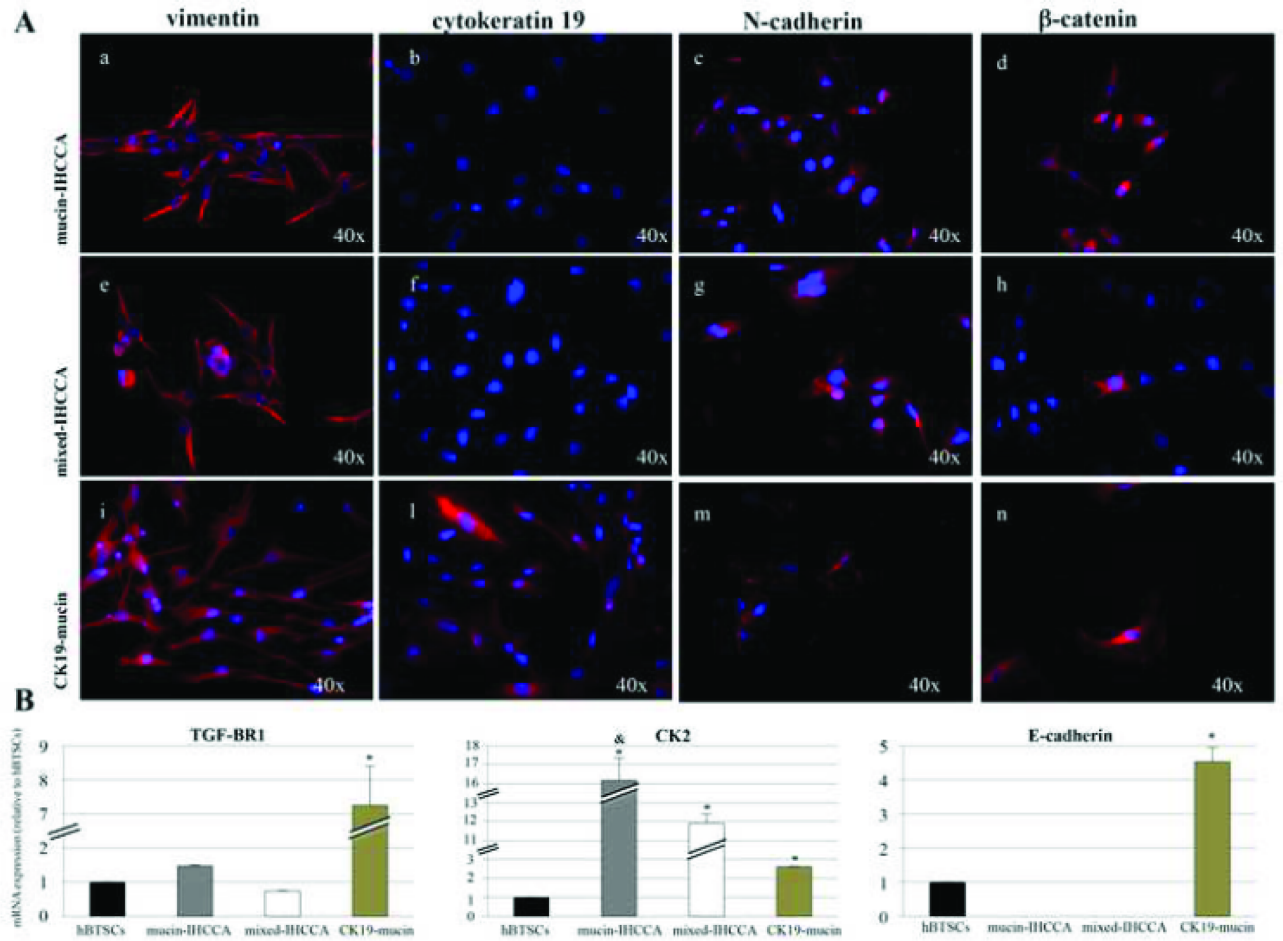
Immunofluorescence (IF) and RT-PCR analyses of primary cell cultures. **A**: By IF, vimentin was expressed in mucin- (a, red), mixed-IHCCA (e, red) and CK19-mucin (i, red) primary cell cultures. CK19 was only expressed in CK19-mucin primary culture (l, red). N-cadherin was expressed in all three primary cultures (c,g and m, red). β-catenin has nuclear and cytoplasmic localization in primary cultures (d,h and n, red). **B:** By RT-PCR, CK2 was more expressed in mucin- and mixed-IHCCA than in CK19-mucin cell cultures or hBTSCs while TGF-ΒR1 and E-cadherin genes were more expressed in CK19-mucin. The E-cadherin gene was virtually unexpressed in mucin- and mixed-IHCCA cell cultures. mRNA expression levels were normalized with respect to hBTSCs, considered equal to 1. * = p<0.05 vs other cell cultures; & = p<0.05 vs mixed-IHCCA. Mean +/- SD of N = 3–5 independent experiments.

Most primary cell cultures, prepared from human mucin- and mixed-IHCCA specimens, stably expressed mesenchymal and EMT markers, namely vimentin, N-cadherin and nuclear catenin (Fig 1), while the epithelial markers CK19 (Fig 1B and 1F) and E-cadherin (data not shown) were not expressed. Interestingly, a limited number of primary cell cultures prepared from surgical specimens of human mucin-IHCCA expressed the epithelial marker CK19 in, on average, 45% of the cells (Fig 1l) (CK19-mucin IHCCA), other than mesenchymal and EMT markers (Fig 1I, 1M and 1N). None of the cell cultures prepared from mixed-IHCCA expressed the epithelial markers CK19 and E-cadherin.

The expression of TGF-ΒR1 and CK2 was evaluated by RT-PCR in primary cell cultures and in human biliary tree stem cells (hBTSCs) used as non neoplastic control cells. The TGF-ΒR1 gene was more expressed in CK19-mucin than in mucin- and mixed-IHCCA cell cultures or hBTSCs (Fig 1B) and its expression correlated with the expression of the E-cadherin gene (r = 0.75, p< 0.02) which predominated in CK19-mucin cultures (Fig 1B). The E-cadherin gene was unexpressed in mucin- and mixed-IHCCA cell cultures. On the contrary, CK2 gene expression predominated in mucin- and mixed-IHCCA cell cultures (Fig 1B, p< 0.05 vs CK19-mucin or hBTSCs).

As previously detailed [5, 19, 21], our primary cell cultures were negative for markers of hematopoietic cells (CD45), tumor-associated macrophages (CD163), activated hepatic stellate cells (GFAP), endothelial cells (CD31), fibroblast-activation protein (FAP), and stromal-derived factor (SDF1).

### Effect of TGF-ΒR1 and CK2 inhibitors on cell viability and apoptosis

**Fig 2:**
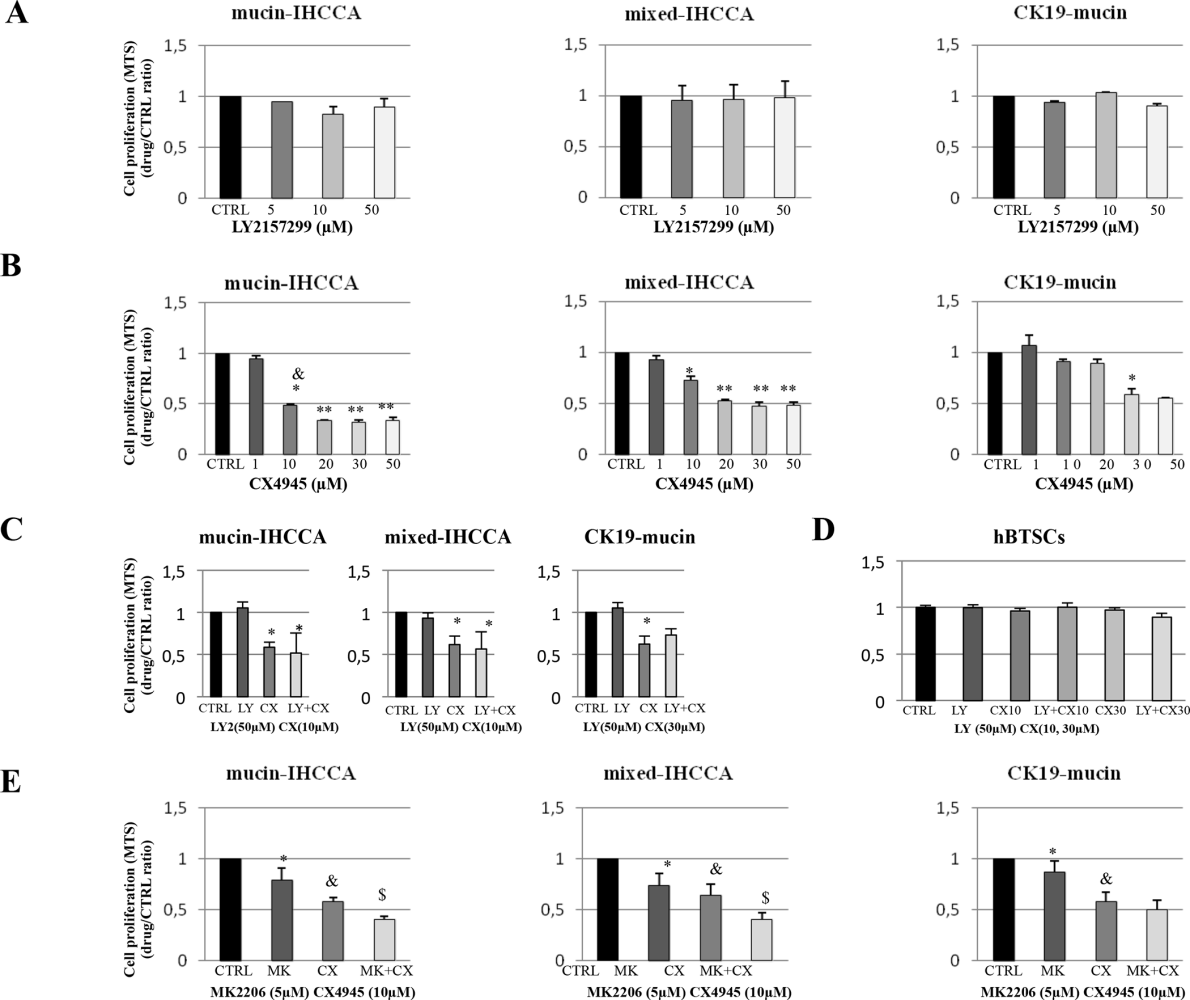
Effect of TGF-ΒR1 inhibitor (LY2157299) and CK2 inhibitor (CX4945) on cell viability (MTS assay). Primary cell cultures were treated with CX4945, LY2157299/5 or MK2206 and MTS was performed at 72 hrs. **A:** TGF-ΒR1 inhibition by LY2157299 did not influence cell viability in any type of primary cell culture. **B:** CK2 inhibition by CX4945 significantly inhibited cell viability in both mucin- and mixed-IHCCA cell cultures starting at a 10 μM concentration, with a more pronounced effect in mucin-IHCCA cells. In CK19-mucin cell cultures expressing high levels of epithelial markers (CK19-mucin and E-cadherin mRNA), the anti-survival effect of CX4945 was observed starting at 30 μM. * = p<0.05 vs other cell cultures;** = p< 0.01 vs 10μM; & = p<0.001 vs 10μM in mixed-IHCCA. **C:** The combination of CX4945 + LY2157299 failed to increase the inhibitory effect of CX4945 on viability of mucin- and mixed- IHCCA cell culture. * = p<0.05 vs control and LY2157299. **D:** LY2157299 and CX4945 treatment, either alone or in combination, did not affect hBTSCs survival. **E:** The combination of CX4945+MK2206 (AKT inhibitor) showed additive inhibitory effects on viability of mucin- and mixed-IHCCA with respect to a single treatment; this was not observed in CK19-mucin. * = p< 0.05 vs controls; & = p<0.05 vs control and MK; $ = p<0.05 vs MK and CX alone. Mean +/- SD of N = 3–5 independent experiments.

Primary IHCCA cell cultures were exposed for 72 hrs to an increasing concentration of the TGF-ΒR1 inhibitor, LY2157299, and cell viability was assessed by MTS assay. Inhibition of TGF-ΒR1 did not affect cell viability in any of the different primary cell cultures investigated; mucin-, mixed-IHCCA, or CK19-mucin (Fig 2A).

CX4945, a potent and selective CK2 inhibitor, reduced cell viability (MTS assay) starting at 10 μM in mixed- and mucin-IHCCA cell cultures and at a 30 μM concentration in CK19-mucin (Fig 2B; mucin-IHCCA IC_50_ = 6.7 +/- 0.001 μM; mixed-IHCCA IC_50_ = 10.5 +/- 0.002 μM; CK19-mucin IC50 >50μM). However, the anti-survival effect of 10 μM CX4945 was significantly higher in mucin- than mixed-IHCCA primary cell cultures (p< 0.05). In substance, the inhibitory effect of CX4945 on cell viability correlated with CK2 expression (mucin- > mixed-IHCCA > CK19-mucin).

Exposure of IHCCA cell cultures to LY2157299+CX4945 did not enhance the inhibitory effect of CX4945 alone on cell viability (MTS assay, Fig 2C). Interestingly, LY2157299 and CX4945 treatment alone or in combination did not affect the viability of hBTSCs (Fig 2D).

The inhibitory effect of CX4945 (CK2 inhibitor) was enhanced by contemporary targeting of the PI3kinase/AKT pathway. The PI3K/AKT signaling is up-regulated in CCA and, recently, we demonstrated the anti-survival and pro-apoptotic effect of the AKT inhibitor (MK2206) on primary IHCCA cell cultures [5]. By combining CK2 and AKT inhibitors (CX4945 and MK2206), we demonstrated an additive inhibitory effect on cell viability (MTS assay) but this occurred only in mucin- and mixed-IHCCA, not in CK19-mucin cell cultures, suggesting a particular resistance of CK19 expressing CCA cells to CK2 and AKT inhibition (Fig 2E).

**Fig 3:**
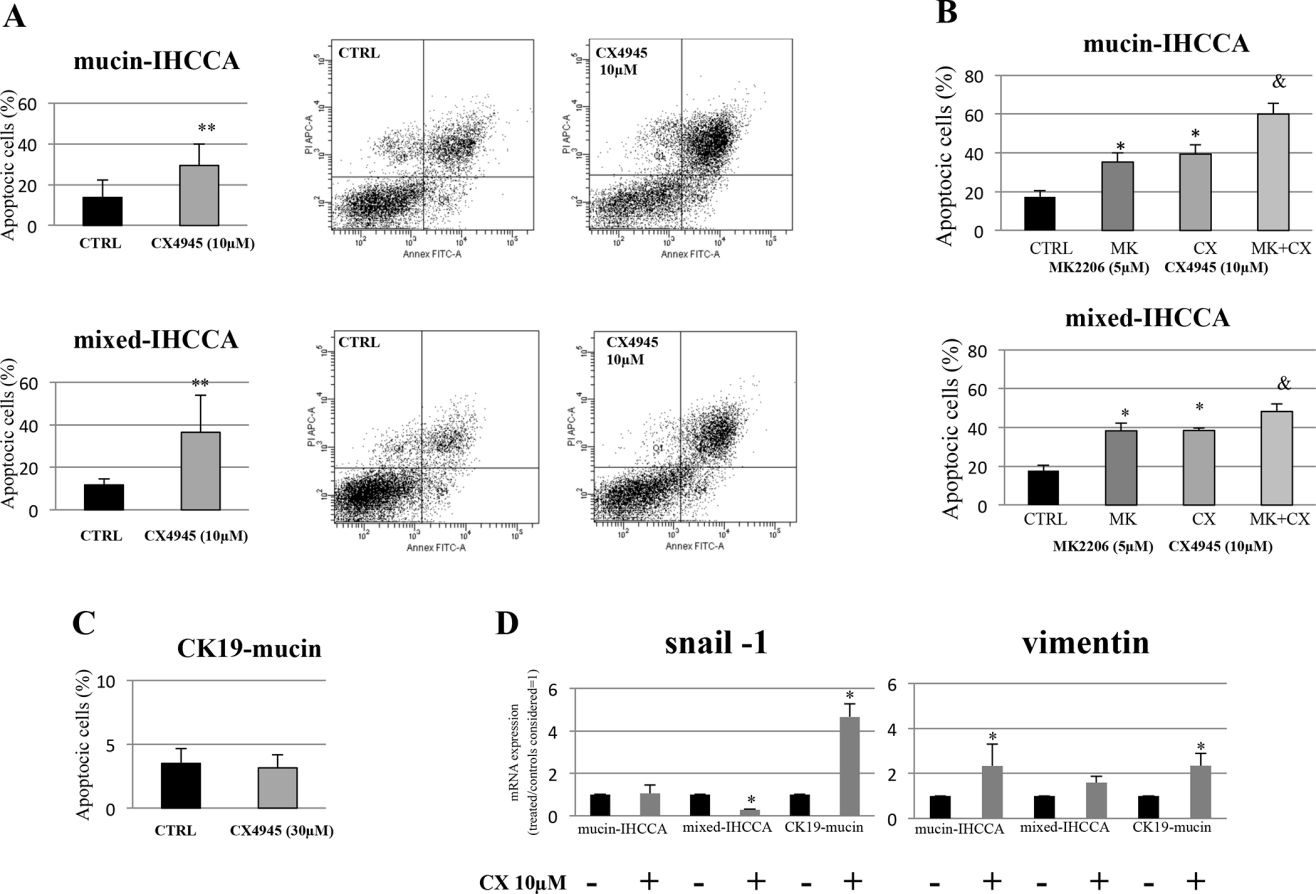
Apoptosis (Annexin V-FITC/PI by FACS) of primary cell cultures exposed to TGF-ΒR1 inhibitor (LY2157299) and CK2 inhibitor (CX4945), and RT-PCR analysis of snail-1 and vimentin in primary cell cultures exposed to CX4945. **A:**After 72 hrs of treatment, CX4945 10 μM induced apoptosis in both mucin- and mixed-IHCCA primary cell cultures ** = p<0.01 vs control. **B:**When CX4945 was combined with MK2206, an enhanced number of apoptotic cells was seen with respect to CX4945 or MK2206 alone. * = p<0.05 vs control and MK+CX; & = p<0.05 vs MK and CX alone. **C:** CX4945 30 μM did not induce apoptosis in CK19 mucin primary cells. **D:** CX4945 (10μM) treatment for 72h induced vimentin expression in all three primary cell cultures, whereas snail-1 expression was induced only in CK19-mucin, being down-regulated in mixed-IHCCA and unaffected in mucin-IHCCA. mRNA expression was normalized to control cells exposed to CX4945 carrier and considered equal to 1. * = p<0.05 vs control. Mean +/- SD of N = 3–5 independent experiments.

Inhibition of TGF-ΒR1 by LY2157299 showed no effect on apoptosis. In detail, the percent of Annexin V positive were unchanged after treatment with LY2157299 of mucin-IHCCA (18+/- 1,02% vs 17,3+/- 1,58% in controls, N = 5) and mixed-IHCCA (11+/- 2,73% vs 12+/- 1,65 in controls, N = 5). In contrast, inhibition of CK2 by CX4945 markedly enhanced apoptosis of IHCCA cell cultures as evidenced by the Annexin V/PI assay. Indeed, after 72 hrs of treatment with CX4945 10μM, annexin V positive cells were significantly enhanced (Fig 3A) in mucin- and mixed-IHCCA cell cultures (p< 0.05 vs controls). The combination of CX4945 and MK2206 increased the apoptotic effects induced by a single treatment in mucin-IHCCA (60+/- 2% vs 36+/- 1.8% for MK and 40+/- 1.5% for CX) and mixed-IHCCA (48+/- 1.4% vs 39+/- 1.7% for MK and 39+/- 0.5% for CX) cell cultures (Fig 3B). Consistent with the higher resistance against CX4945 anti-proliferative effects, CK19-mucin cell cultures showed no increase of apoptosis when exposed to the CK2 inhibitor (Fig 3C).

A number of recent evidence suggests that chemo-resistance is caused by induction of the EMT program [22]. To explore cell mechanisms of resistance against CX4945 we examined EMT markers snail-1 and vimentin in our primary cell cultures exposed (72hrs) to CX4945 treatment (Fig 3D). RT-PCR indicated that CX4945 induced vimentin expression levels in all three primary cell cultures despite in mixed-IHCCA in a non-significant manner while snail-1 over-expression was evident only in CK19-mucin cells (Fig 3D). These results indicate that CK19-mucin IHCCA cells underwent more significant phenotypic changes with respect to mucin- and mixed-IHCCA (via EMT) when treated with CX4945. This is consistent with the resistance of CK19-mucin IHCCA cells against CX4945 and, in particular, against the pro-apoptotic effect of this drug which are somehow linked with the induction of the EMT trait. In contrast, mucin- and mixed-IHCCA where snail-1 was not induced by CX4945 were sensitive to the apoptotic effect of the drug.

**Fig 4:**
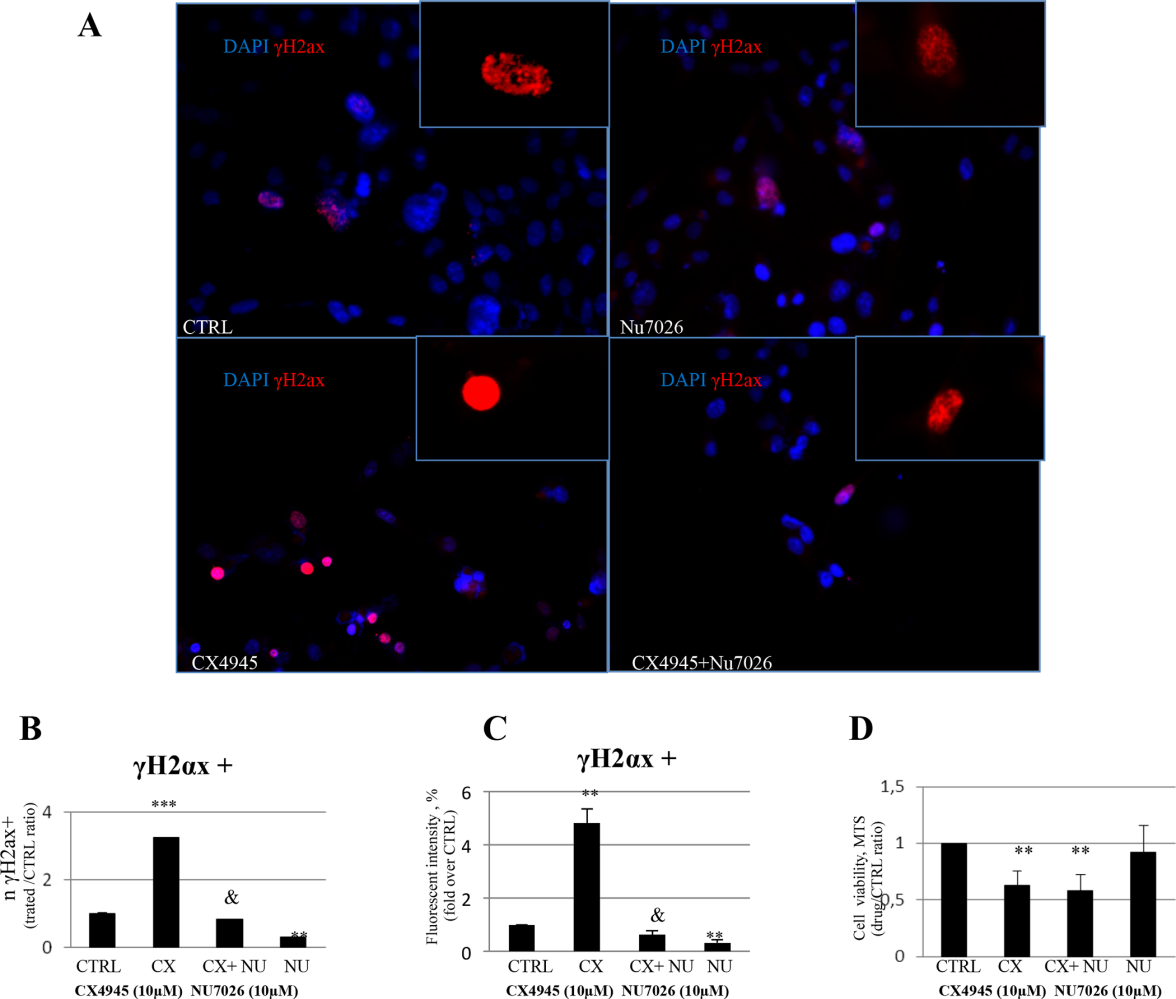
Immunofluorescence for γH2ax and MTS assays. **A-C:** mucin-IHCCA primary cell cultures were investigated by IF for γ-H2ax nuclear foci, a biomarker of DNA double-strand breaks. After 72hrs of treatment, CX4945 (10μM) increased the number of γ-H2ax positive (red) nuclei vs control. A potent DNA-PK inhibitor (NU7026, 10 μM) reduced γ-H2ax positive nuclei as indicated by fluorescent intensity vs controls and blocked the effects of CX4945 on γ-H2ax ** = p<0.01 vs other columns;*** = p<0.001 vs other columns; & = p<0.01 vs CX4945 and NU7026 alone. **D**: NU7026 alone or in combination with CX4945 failed to influence cell viability in mucin-IHCCA cultures vs control and CX4945 alone.** = p<0.01 vs control and NU7026 alone. Mean +/-SD of N = 3–5 independent experiments.

The protein kinase CK2 regulates several components of DNA-repair/DNA-damage sensing machinery, including XRCC 1 and 4, Rad9 and DNA-PK (DNA-dependent protein kinase) [23–25]. Immediately after the formation of DNA double-strand breaks, CK2 phosphorylates DNA-PK and then positively modulates H2AX which is used as a biomarker of DNA double-strand breaks. With this in mind we used selective CK2 and DNA-PK inhibitors to evaluate whether the anti-proliferative and pro-apoptotic effects of CK2 inhibition involve DNA-PK and its target H2AX in CCA. The CK2 inhibitor, CX4945, in association with anti-survival and pro-apoptotic effects increased, in mucin-IHCCA primary cultures, the number of γ-H2ax nuclear foci, identified by IF (Fig 4A and 4B). We analyzed mucin-IHCCA primary cultures as representative cells responsive to CX4945. This suggests an active involvement of CK2 in DNA repair in CCAs. Contrary to what we expected, the DNA-PK inhibitor NU7026 reduced the number of y-H2AX-positive cells compared to control, without influencing cell viability (Fig 4A, 4B, 4C and 4D). Most importantly, NU7026 treatment was able to rescue the increased number of γ-H2ax nuclear foci induced by the CK2 inhibitor, CX4945 (Fig 4), suggesting that CK2 and DNA-PK in the setting of CCA act as antagonists in the modulation of DNA repair machinery.

### Effect of TGF-ΒR1 and CK2 inhibitors on cell migration

**Fig 5:**
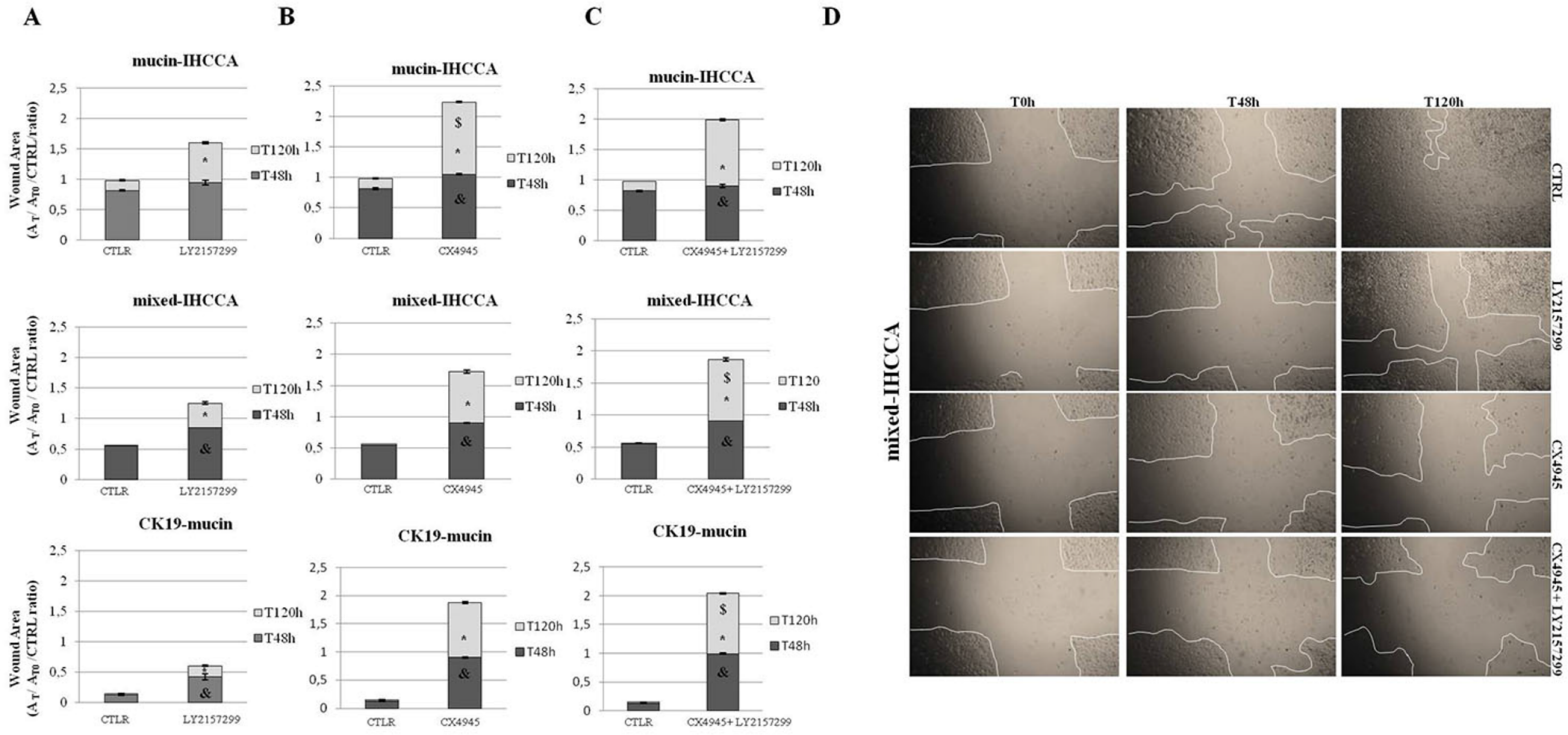
Wounding-healing assays. Mucin-IHCCA, mixed-IHCCA and CK19-mucin cell cultures were treated with LY2157299 (50 μM), CX4945 (10μM) or LY2157299 (50 μM) + CX4945 (10μM). **A:** pharmacological inhibition of TGF-ΒR1 by LY2157299 produced inhibitory effects on cell migration. In mucin-IHCCA the effects of LY2157299 were evident at 120 hrs, while in mixed-IHCCA and CK19-mucin effects were already visible at 48hrs. & = p<0.05 vs CTLR;* = p<0.05 vs CTLR. **B:** CX4945 exerted more evident inhibitory effects on cell migration in mucin-IHCCA and the effects were higher with respect to LY2157299. & = p<0.05 vs CTLR and LY2157299 alone; * = p<0.05 vs CTLR **and** LY2157299 alone; $ = p< 0.05 vs mixed- and CK19-mucin. **C:** the combination of CX4945 andLY2157299 exerted relatively more evident additive effects on cell migration in mixed-IHCCA than in CK19-mucin, but no additive effect was seen in mucin-IHCCA. & = p<0.05 vs CTLR;* = p<0.05 vs CTLR; $ = p< 0.05 vs CX4945 alone. **D**: representative images of mixed-IHCCA cell cultures where the synergistic effect of CX4945+LY2157299 was more evident with respect to the other cell lines. The wound area was calculated with respect to control at T0, considered as 1. Mean +/- SD of N = 3–5 independent experiments.

TGF-b induces migration of cancer cells by activating N-Cadherin and inducing Twist and vimentin [16] with consequent morphological cell changes [16, 26]. Therefore, we tested the effects of TGF-ΒR1 and CK2 inhibitors on cell migration, evaluated by the wound-healing assay (Fig 5, S1 Fig and S2 Fig). Stretches were performed in monolayers of IHCCA primary cultures, and wound healing was assessed after treatment with LY2157299 (50 μM), CX4945 (10μM or 30 μM) or with the combination of CX-4945 (10 μM or 30 μM) and LY2157299 (50μM). LY2157299 impaired the migration of mixed-IHCCA and CK19-mucin at both 48 and 120 hrs and of mucin-IHCCA at 120 hrs (Fig 5A and 5D). CX4945 showed a stronger inhibitory effect on cell migration in mucin-IHCCA than in mixed-IHCCA or CK19-mucin (p< 0.05, Fig 5B and 5D) and the inhibitory effects were also significantly higher with respect to LY2157299 (p< 0.05, Fig 5B and 5D). The combination of LY2157299 plus CX4945 exerted relatively more evident additive inhibitory effects on cell migration in mixed-IHCCA (Fig 5C and 5D, p< 0.05 vs CX4945 alone) than in CK19-mucin (p< 0.05 vs CX4945 alone), but was without effect in mucin-IHCCA (Fig 5C and 5D).

**Fig 6:**
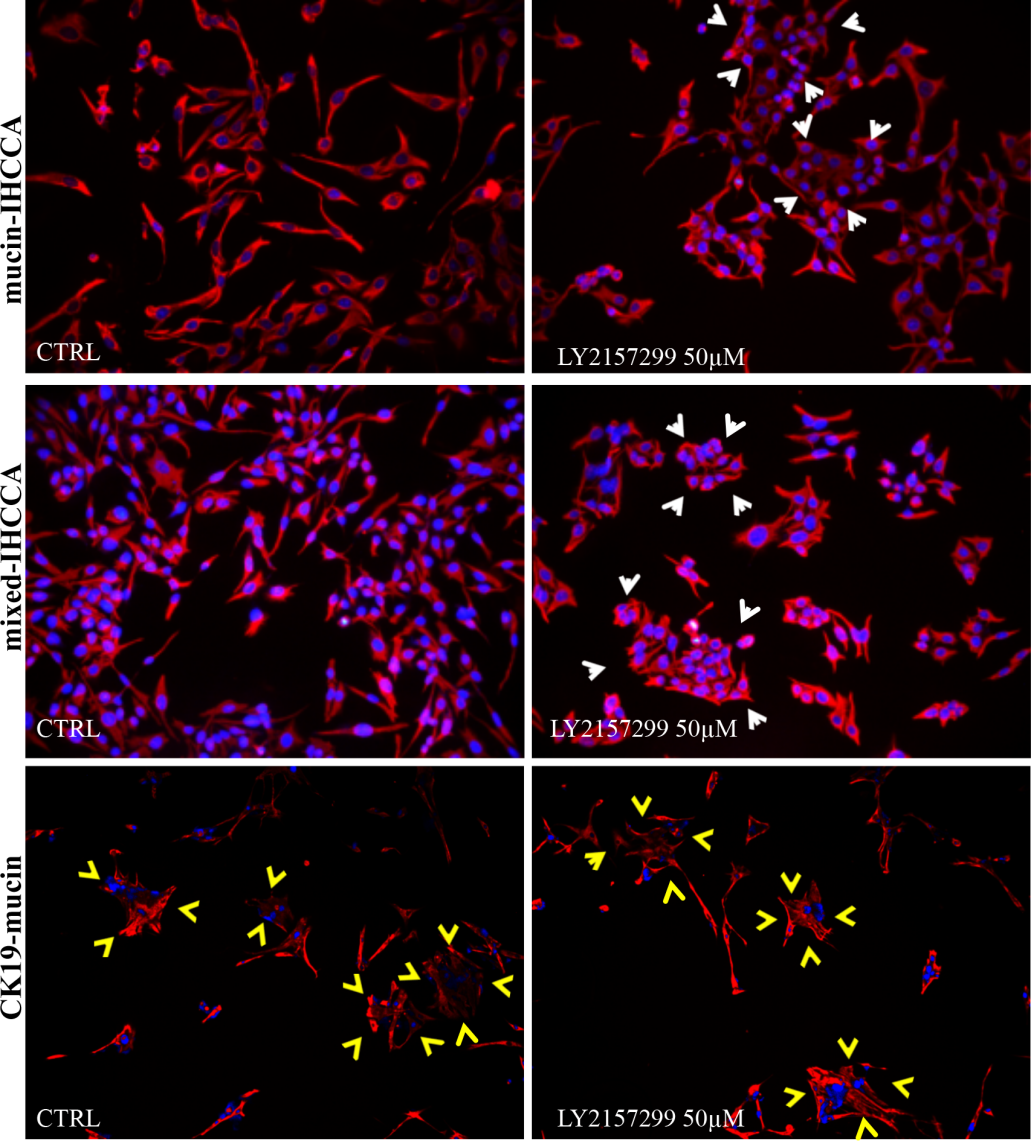
Immunofluorescence for vimentin in cell cultures treated with the TGF-ΒR1 inhibitor LY2157299. Expression of vimentin was unchanged by LY2157299. However, IF for vimentin (red) highlighted evident morphological changes in mucin- and mixed-IHCCA cell cultures treated for 72 hrs with the TGF-ΒR1 inhibitor LY2157299 (50 μM). LY2157299 induced the loss of typical mesenchymal features, instead favoring the epithelial phenotype with formation of cell clusters (white arrows). No morphologic change was induced by LY2157299 in CK19-mucin cell cultures. Cell clusters with undefined edges, a typical feature of the epithelial phenotype, were observed in mucin- and mixed- IHCCA cells treated with LY2157299 (white arrows) and in CK19-mucin (yellow arrows, similar in treated and untreated cells). Mean +/- SD of N = 3–5 independent experiments.

To evaluate whether the impaired cell migration induced by the TGF-ΒR1 inhibitor LY2157299 was associated with a decreased expression of vimentin [27], we investigated, by IF, vimentin expression in cell cultures incubated for 72 hrs with LY2157299. IF for vimentin showed no significant changes, indicating that the impairment of migration induced by LY2157299 is probably vimentin-independent (Fig 6). However, these experiments revealed a significant phenotypic change of mucin- and mixed-IHCCA cultures (Fig 6) where LY2157299 induced the loss of typical mesenchymal features, instead favoring the formation of cell clusters with undefined edges, a typical feature of the epithelia phenotype [16, 28] (Fig 6, white arrows). Control cells maintained features of scattered colonies with scarce intercellular contacts and CK19-mucin cells showed no morphological changes during incubation with LY2157299. In substance, cell clusters typical of an epithelial phenotype were observed in CK19-mucin (untreated) and LY2157299-treated cells (Fig 6).

### In vitro tumorogenicity: CX4945 effects on clonogenicity of IHCCA primary cells

Recently, it has been demonstrated that CX-4945 may partially block the PI3K/AKT/mTOR pathway and transiently induce the apoptotic effectors [29].

**Fig 7:**
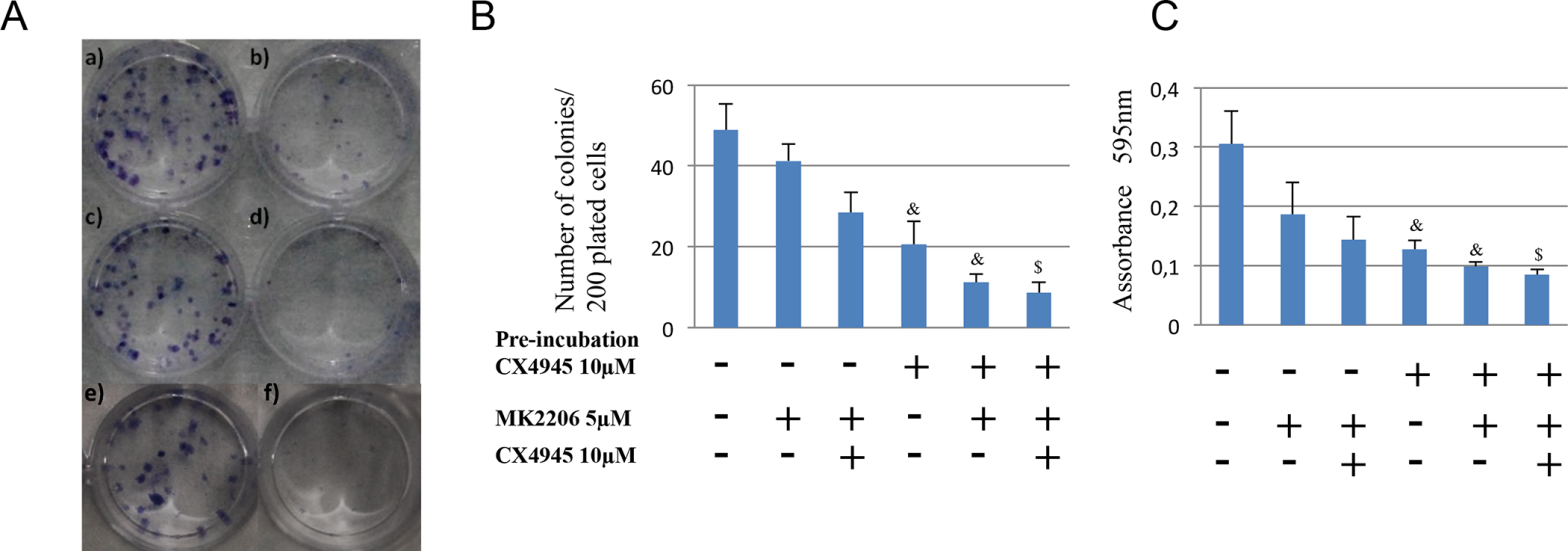
Clonogenic assays. The number of colonies (A and B) and the absorbance of the extracted dye (crystal violet) measured at 595 nm (C) indicate the clonogenic potential of CCA cell cultures. We tested the clonogenic capacity of mucin-IHCCA cells after sequential treatment with CX4945 (72 hrs) and MK2206 (48 hrs) versus their combination. The sequential treatment of CX4945 followed by MK2206 showed the highest inhibitory effect on mucin-IHCCA cell growth. We calculated the numbers of colonies and the absorbance of the extracted dye at 595 nm. & = p<0.05 vs non pre-incubated conditions. $ = p< 0.05 vs others columns. Mean +/- SD of N = 3–5 independent experiments.

As tested by the clonogenic assay, a well established method of in vitro tumorigenicity [30], we demonstrated that incubation for 72h with CX4945 strongly reduced the ability of mucin-IHCCA to form colonies (Fig 7A–7C). Since CX4945 plus the AKT inhibitor MK2206 showed additive effects on apoptosis in mucin-IHCCA, we tested whether a sequential treatment with CX4945 followed by MK2206 further increases the inhibitory effect on colony formation. Fig 7 showed that the sequential treatment induced a stronger effect than the two drugs combined (Fig 7A–7C). Our results suggest that MK2206 overcomes the weak resistance of IH-CCA cells toward CX4945, strongly reducing the colony formation capacity vs the combined treatment of CX4945 + MK2206 (Fig 7B).

### Mucin-IHCCA primary cell cultures resistant to CX4945 treatment showed deactivation of EGFR (epidermal growth factor receptor) and HGFR (hepatocyte growth factor receptor)

Both EGFR and HGFR are involved in CCA progression and prognosis [31]. Recent studies showed that CX4945 induced down-regulation of the autophagosome-mediated EGFR in lung cancer [32], while EGFR inhibitors induced, in lung adenocarcinoma, activation of the HGF/Met pathway as a mechanism of drug resistance [33].

**Fig 8:**
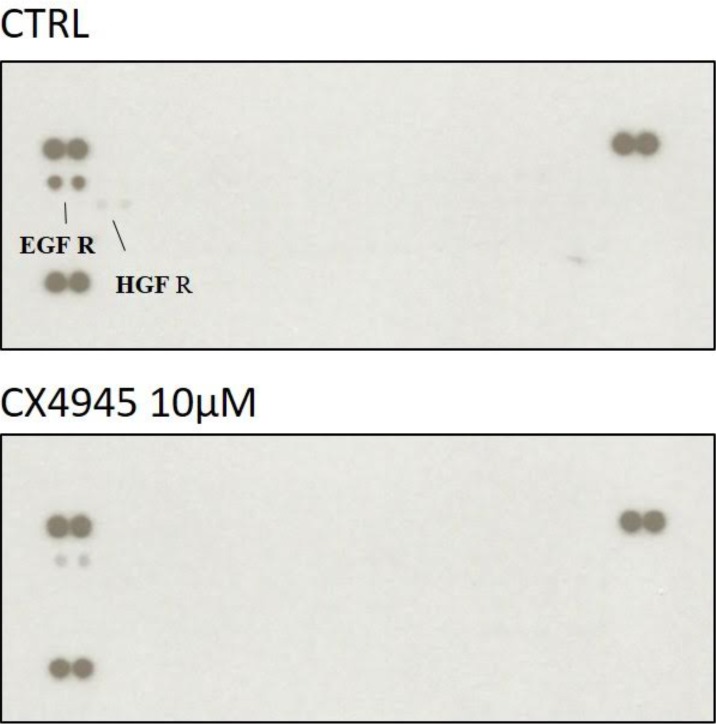
RTK human phosphoarrays in mucin-IHCCA cells resistant to CX4945 treatment. After 72h of CX4945 (10 μM) treatment, surviving cells showed contemporary deactivation of both EGFR and HGFR signalling. The black lines indicate the spots positive for EGFR and HGFR activation.

To evaluate whether EGFR and/or HGFR related pathways are involved in the resistance of mucin-IHCCA toward CX4945, we evaluated their phosphorylation under CX4945 treatment. Our results (Fig 8) revealed that CX4945-resistant cells display down-regulation of both EGFR and HGFR (Fig 8) and, therefore, that alternative pathways are involved in such resistance.

## Discussion

In the last few years, research on cancer treatment has shifted from cytotoxic, non-specific chemotherapeutic drugs to molecular targeted, rationally designed therapies [30]. Therefore, defining the pathways involved in cancer initiation and progression is crucial in order to generate molecules which are able to selectively hit cancer cells without affecting normal cells and drug induced reactions. CCA comprises a heterogeneous group of cancers highly resistant to chemotherapeutics [1]. The standard of care for non-surgically treatable CCA is the gemcitabine/cisplatin combination, which ensures only a few months of improved survival [34]. A number of different targeted therapies have been tested in pilot trials without evident benefit. Different mechanisms have been evoked to explain the chemo-resistance of CCA including the abundance of CSCs and the predominance of EMT traits which have been associated with aggressiveness, spreading and metastasis [1]. Therefore, in CCA as in other cancers, EMT represents a crucial biologic target for therapies. TGF-β pathways are up-regulated in CCA [17] and play a central role in driving EMT [16, 17, 26, 15]. Exogenous stimulation of TGF-β related pathways induce morphological changes of CCA cell lines typical of EMT (26) and indeed our group has recently highlighted the relevance of EMT in *in vivo* and in *vitro* studies [5]. Specifically, we demonstrated that in specimens of human IHCCAs, 30% of CCA cells co-express mesenchymal and epithelial markers and EMT traits *in situ*. Moreover, i*n vitro*, primary human IHCCA cell cultures prepared from human IHCCA specimens showed an abundance of CSCs which correlate with EMT trait markers and which could be a main determinant of the typical desmoplastic nature of this cancer [5].

In the present study, we tried to pharmacologically target the TGF-β pathway and related signalling to modulate EMT in order to provide pre-clinical evidence of potentially successful therapies for IHCCA. The study was performed in primary cell cultures prepared from specimens of human IHCCA subtypes, as previously described [5].

TGF-ΒR1 was expressed in all primary culture subtypes, both the ones expressing only mesenchymal markers (vimentin, N-cadherin, nuclear β–catenin) or those maintaining the expression of epithelial marker CK19. Interestingly, TGF-ΒR1 expression was higher in CK19-mucin than in cell cultures lacking epithelia marker expression, while the opposite occurred for CK2 expression. The specific and selective TGF-ΒR1 inhibitor LY2157299 failed, in our experiments, to influence cell viability or apoptosis. Rather, it impaired cell migration, an effect seen in all tested primary cell cultures and without changing the vimentin expression (IF). Notably, the inhibitory effects of LY2157299 on migration were associated with the acquisition of typical epithelial features. In keeping with our results, in a hepatocellular carcinoma (HCC) cell line, LY2157299 blocked cell migration by activating SMAD2, but independently from TGF-b receptors (TGF-bRs) [35]. Most importantly, LY2157299 is currently under investigation in a phase II clinical trial recruiting HCC patients (NCT01246986, http://clinicaltrials.gov).

We then tested CX4945, a drug targeting CK2, a player strictly linked with TGF-β-related pathways, which has been recently investigated in a phase I trial for multiple myeloma and in a phase II clinical trial of different solid tumors [36]. Recently, a cross-talk between CK2 and TGF-β signaling was demonstrated during the evolution of the EMT process [37]. Apart from the modulation of TGF-β signaling, CK2 exerts pleiotropic effects on cancer cells [38–40] including phosphorylation of Akt [39] and regulation of DNA-repair machinery [23–25]. We demonstrated a significant effect of CX4945 on both viability and apoptosis, these effects being strictly correlated with the expression levels of CK2 in our primary cell cultures, mucin->mixed-IHCCA> CK19-mucin. Consistently, in CK19-mucin, which expresses low CK2 levels, no apoptotic effects (only anti-survival action) were induced by CX4945. In breast cancer also, the CK2 expression level correlates with the anti-proliferative effects induced by CX4945 [28]. In the A549 non-small cell lung cancer cell line, the anti-proliferative effects are TGF-β-dependent and CX-4945 inhibits the TGF-β1-induced cadherin switch [41]. In colorectal cancer, CK2a modulates cell proliferation and invasion by regulating EMT-related genes [38]. The IC_50_'s for CX-4945 anti-proliferative effects in squamous cell carcinoma cell lines, measured by MTT assay, ranged from 3.4–11.9 μM [29], while in lymphoma cells the maximal apoptotic effect was observed at 10 μM [29] and this is in line with our results.

Interestingly, our data showed that the CK2 inhibitor, CX4945, increased the number of γ-H2ax nuclear foci in mucin-IHCCA primary cultures, the cell culture more responsive to CX4945, suggesting an active involvement of CK2 in DNA repair in CCAs. In parallel, we demonstrated an inhibitory effect of CX4945 on cell migration consistent with the anti-survival and pro-apoptotic effects of the drug. Since the TGF-ΒR1 inhibitor failed to influence the effects of CX4945, it is evident that the involvement of CK2 in CCA cell viability is independent from TGF-ΒR1, justifying the proposal to test in clinical trials the combination of the two inhibitors (CX4945 + LY2157299).

Since AKT is activated by CK2 [32] and AKT inhibitors are effective in vitro against CCA [5], we tested the combination of MK2206/CX4945, demonstrating, at least in mucin- and mixed-IHCCA, additive effects on apoptosis.

In substance, our results indicate that both TGF-β and CK2 pathways are involved in CCA cell viability and migration, thus supporting the rationale for testing selective inhibitors in clinical trials. Specifically, the combination of TGF-β, CK2 and/or AKT inhibitors, proven to be safe in the clinic, could exert beneficial effects on CCA progression. Therefore, our results have a translational value in suggesting a clinical investigation combining inhibitors of TGF-ΒR1 and CK2 as treatment for IHCCA. Furthermore, the fact that many of these compounds are already in clinical phases for different cancers should represent an advantage.

## Supporting information

S1 Fig Wounding-healing assays. IHCCA-mucin cell cultures were treated with LY2157299 (50 μM), CX4945 (10μM) or LY2157299 (50 μM) + CX4945 (10μM).
(JPG)

S2 Fig Wounding-healing assays. CK19-mucin cell cultures were treated with LY2157299 (50 μM), CX4945 (10μM) or LY2157299 (50 μM) + CX4945 (10μM).
(JPG)
