# Supplementary figures and images for: TGF-β signaling is an effective target to impair survival and induce apoptosis of human cholangiocarcinoma cells: A study on human primary cell cultures
Source: PLoS One. 2017 Sep 5;12(9):e0183932. doi: 10.1371/journal.pone.0183932 (PMC5584931; doi:10.1371/journal.pone.0183932)

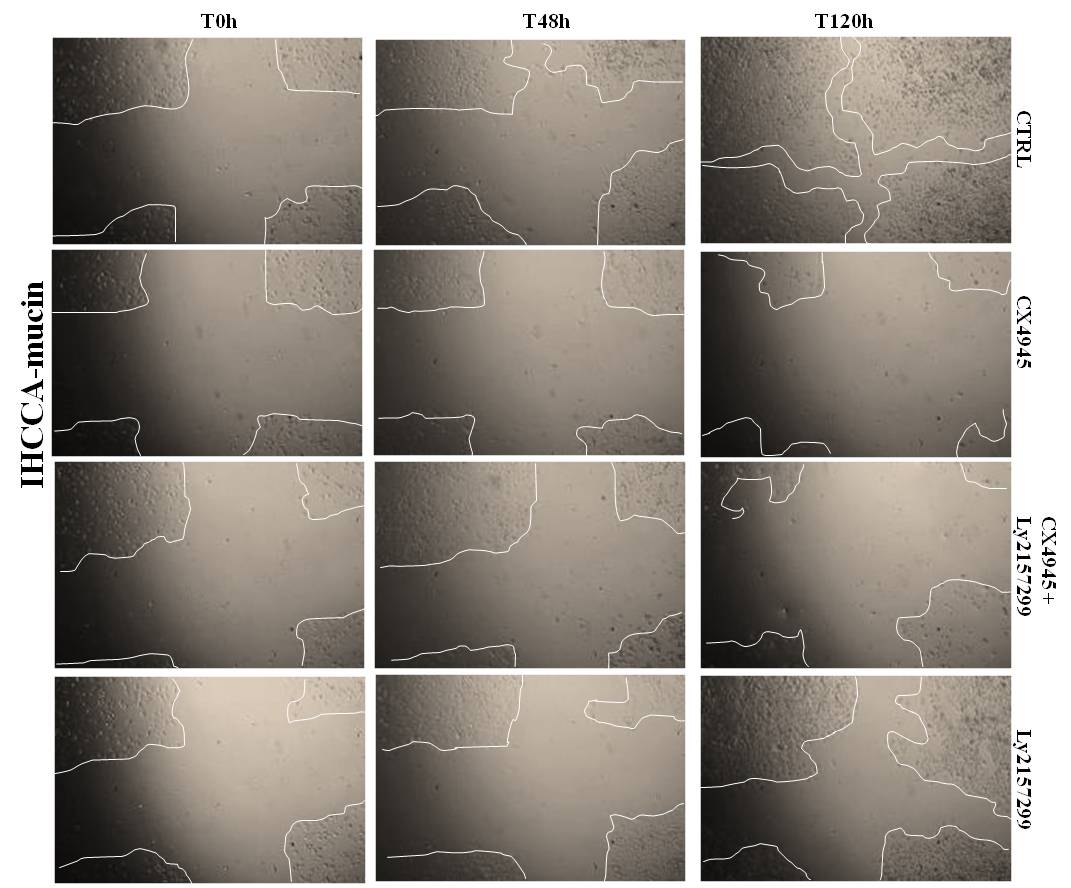

Supplement: S1 Fig — IHCCA-mucin cell cultures were treated with LY2157299 (50 μM), CX4945 (10μM) or LY2157299 (50 μM) + CX4945 (10μM). (JPG) [file pone.0183932.s001.jpg]

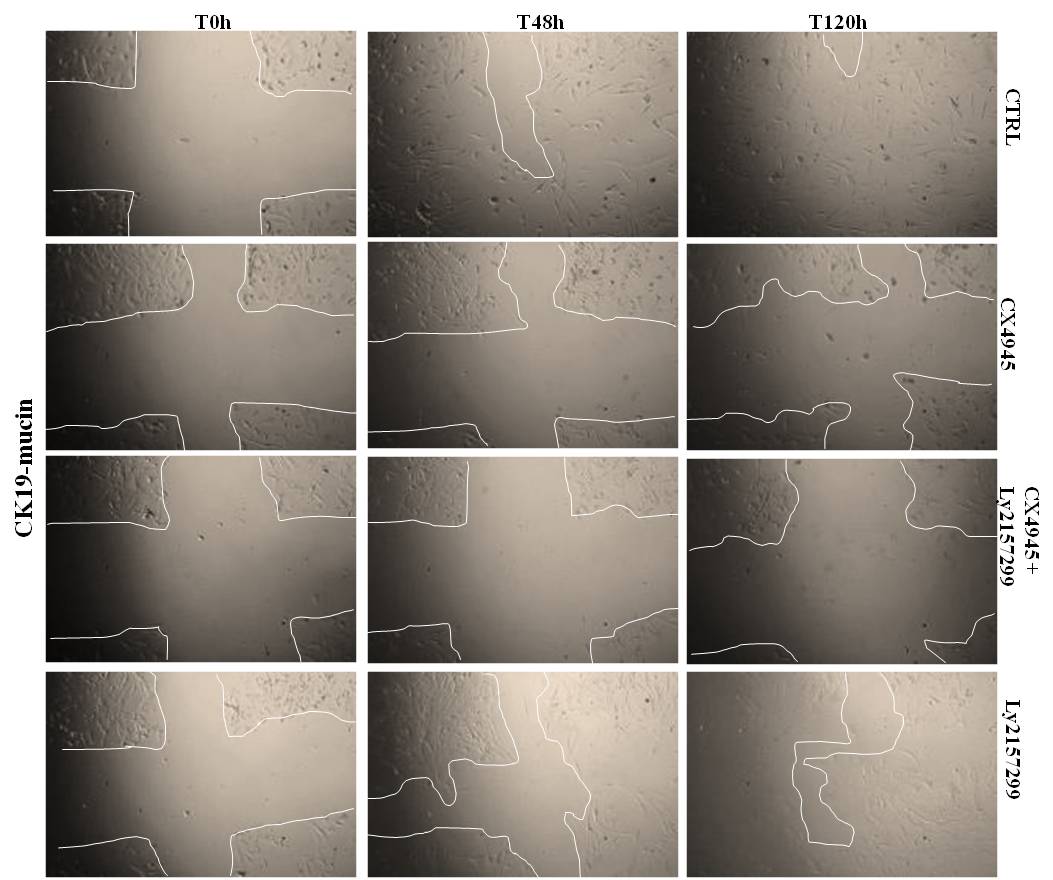

Supplement: S2 Fig — CK19-mucin cell cultures were treated with LY2157299 (50 μM), CX4945 (10μM) or LY2157299 (50 μM) + CX4945 (10μM). (JPG) [file pone.0183932.s002.jpg]
